# Association between Dietary Total Vitamin A, β-carotene, and Retinol Intake and Risk of cardiometabolic multimorbidity: Results from the China Health and Nutrition Survey, 1997–2015

**DOI:** 10.21203/rs.3.rs-4384704/v1

**Published:** 2024-05-30

**Authors:** Yudi Tang, Yao Xiao, Fen Yang, Xiaolian Gao, Xinhong Zhu, Guiyuan Qiao

**Affiliations:** Hubei University of Chinese Medicine; Hubei Provincial Hospital of Traditional Chinese Medicine; Hubei University of Chinese Medicine; Hubei University of Chinese Medicine; Hubei University of Chinese Medicine; Hubei University of Chinese Medicine

**Keywords:** vitamin A, β-carotene, retinol, Cardiometabolic multimorbidity, Prospective cohort study

## Abstract

**Background:**

The association between vitamin A and single cardiometabolic diseases has been extensively studied, but the relationship between dietary vitamin A intake and the risk of cardiometabolic multimorbidity (CMM) has not been studied. Therefore, the present study was conducted to explore the association with CMM risk by analyzing different sources of vitamin A.

**Methods:**

This study utilized 13,603 subjects aged ≥ 18 years from 1997–2015 from the China Health and Nutrition Survey (CHNS). Dietary intake was calculated from 3 consecutive 24-h dietary recalls combined with a house hold food inventory. CMM is defined as the development of at least two cardiometabolic diseases.

**Results:**

After a median follow-up of 9.1 years, there were 1050 new cases of CMM. The risk of CMM was significantly lower in those with higher vitamin A intake (Q1 vs Q5 HR 0.66, 95% CI 0.54–0.81). β-carotene (Q1 vs Q5 HR 0.82, 95% CI 0.66–1.02) and retinol (Q1 vs Q5 HR 0.59, 95%CI 0.48–0.73) intake had a similarly negative correlation. Using restricted cubic spline found an L-shaped relationship between retinol intake and CMM (*p* non-linear < 0.001). In subgroup analyses, protective effects were stronger for participants aged ≥ 44 years (HR 0.72, 95%CI 0.57–0.92) and for the female group (HR 0.62, 95%CI 0.45–0.84).

**Conclusion:**

Dietary vitamin A was a protective factor for CMM, and this effect was stronger in age ≥ 44 years and in the female group. There was a ceiling effect on the protective effect of retinol intake on the risk of CMM.

## Introduction

Multimorbidity can be defined as the simultaneous occurrence of at least two diseases in the same person, and it can be a combination of several different chronic diseases^1^. The coexistence of multiple diseases complicates the management of this group of patients, with more comprehensive treatment needs leading to greater consumption of health resources, and prevention and improved management of multimorbidity are now key priorities in many countries^2^. Cardiometabolic multimorbidity (CMM), defined as the coexistence of 2 or more cardiometabolic diseases, including hypertension, diabetes, stroke and cardiovascular disease, is one of the most common multimorbidity patterns^3^. The incidence of CMM has significantly increased over the past two decades with the rise in life expectancy and ongoing advancements in the management of cardiovascular diseases and diabetes^4^. It has been reported that CMM not only significantly increases the risk of adverse outcomes such as dementia, reduces the quality of life of patients, and results in shorter life expectancy, but also poses a significant challenge to the healthcare system^5–9^. In response to this challenge, research on CMM has intensified, and the role of nutrients at the level of modifiable risk and preventive factors has gained widespread attention^10–13^.

Vitamin A refers to the compounds retinal, retinol and its esters, which is an indispensable micronutrient necessary for normal physiological function, plays an important role in cell differentiation, embryonic development, vision and immune function^14–16^. Carotenoids from plants (eg, fruits and vegetables) and retinol from animals (eg, liver, kidneys, dairy products) are the main dietary sources of vitamin A for most people^17,18^. In a prospective study focusing on hypertension, it was pointed out that the total dietary intake of vitamin A showed an L-shaped relationship with the incidence of new-onset hypertension^19^. Subsequently, in a study involving 17,111 participants with a median follow-up time of 11 years, the authors reported an inverse relationship between dietary intake of vitamin A and the risk of diabetes, particularly in men (HR 0.69, 95%CI 0.49–0.97)^20^. A meta-analysis pooling the combined effects of 20 observational studies of risk indicators showed that vitamin A and its organic compounds were negatively associated with the risk of stroke (log OR −0.46, 95%CI −0.81; −0.12)^21^. Thus far, the majority of extensively reported associations have primarily centered on a single disease model, with insufficient credible evidence available for investigating the relationship between dietary intake of vitamin A and CMM.

Therefore, in order to fill the gaps in existing knowledge, we will use the data from the China Health and Nutrition Survey (CHNS), a longitudinal study spanning 18 years, to comprehensively investigate the prospective association between dietary total vitamin A, β-carotene, retinol intake, and CMM, providing important epidemiological evidence for the prevention of CMM at the level of knowledge.

## Methods

### Study population

The CHNS is an ongoing multipurpose longitudinal study that began in 1989–2015 and includes 288 communities in 12 provinces/autonomous municipalities in China, with follow-up visits scheduled every two to four years. According to the 2010 census, the provinces included in the CHNS sample accounted for 47% of China’s population by 2011. The CHNS was established as a joint project of the University of North Carolina at Chapel and the Chinese Academy of Preventive Medicine (now the China Center for Disease Control and Prevention [CCDC]). Each round of surveys collected data on sociodemographic factors, diet, physical activity, health and behavioral changes at the household and individual level in relation to urbanization and social and economic changes at the community level^22^. A detailed description of the methodology of the survey and design is presented in other literature^23–25^.

**Figure 1:**
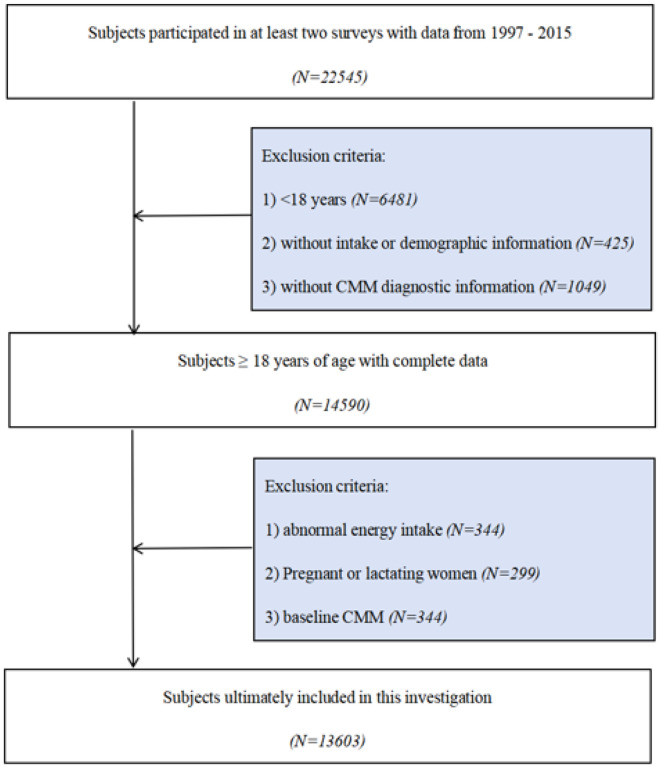
Flowchart of participant selection from the China Health and Nutrition Survey (CHNS) 1997–2015.

This study used data from 1997 to 2015 and included a total of 22,545 subjects who participated in at least 2 surveys. We excluded those aged <18 years (N=6481), without intake or demographic information (N=425), without information on CMM diagnosis (N=1049), abnormal energy intake (<800 or >6000 kcal/d for males and <600 or >4000 kcal/d for females) (N=344), females who were pregnant or breastfeeding (N=299), and baseline those who had CMM (N=344). Finally, a total of 13,603 participants were included in the study (Figure 1).

### Assessment of nutrient intake

In each survey, dietary assessment is conducted face-to-face by trained nutritionists. Individual dietary data are collected using standardized questionnaires, with a 24-hour dietary recall conducted on each of three consecutive days randomly assigned each week. Meanwhile, a combination of weighing and measurement techniques is used to measure household food consumption based on stock changes from the beginning to the end of each day, with dietary intake expressed as per capita daily consumption. The consumption of cooking oil and seasonings by each individual in the household is estimated through weighted household food intake. Previous detailed descriptions of dietary measurements have been published, and the accuracy of dietary assessment methods has been validated^26,27^.

The compositional database for assessing nutrient intake was obtained from the China Food Composition Table (CFCT). In view of the fact that it is often necessary to test for different forms of vitamin A in food and that each form has its own different biological activity, the biological activity of vitamin A is generally expressed in terms of retinol equivalents(RE)^28,29^. The intake of each nutrient is represented by calculating the cumulative average value from the baseline to the last available round before the final round.

### Covariates

In our current study, the covariates selected were factors known or suspected to be associated with the risk of CMM or variables that differed significantly between different sources of vitamin A intake, which were obtained in a structured questionnaire, including age, sex (male/female), district (rural/urban), region (north/south), education (Illiteracy/Primary school/Middle school/High school or above), smoking status (Current smoker/Former/Never), alcohol drinking (no/yes), individual income level (Low/Medium/High/Very high), body mass index (<18.5/18.5–24/24–28/≤28), physical activity (light/moderate/heavy/unknown), and dietary intakes of total energy, protein, carbohydrate, dietary fiber, sodium to potassium ratio, calcium, zinc (all in quintiles).

### Assessment of CMM

The main outcome was CMM, defined as progression to at least two of the following cardiometabolic diseases: hypertension, diabetes, stroke and myocardial infarction. The participant’s disease history was synthesized by responses to the following questions at each follow-up visit: “What was the doctor’s diagnosis of your illness or injury?” “Has a doctor ever told you that you suffer from high blood pressure/diabetes/myocardial infarction/stroke?” “How old were you when the first event occurred?” “Has the event happened in the past year?” “Did you use any of these treatment methods?” “Are you currently taking anti-hypertension drugs?” Among the diagnosis of hypertension are two additional criteria: 1) an average of 3 systolic BP (SBP) measurements≥140 mmHg; 2) an average of 3 diastolic BP (DBP) measurements≥90 mmHg.

### Statistical analysis

The follow-up time is calculated from the start of participants’ involvement in the survey to the diagnosis by CMM, death, or the end of follow-up in these three states, whichever comes first. In all subsequent analyses, participants were divided into five groups according to the quintiles (Q1–Q5) of total vitamin A intake. Continuous variables in baseline characteristics were summarized as mean and standard deviation (SD) or median (IQR), categorical variables were expressed as frequency and percentage, and differences between different quintiles of vitamin A intake were compared using ANOVA tests or Chi-square tests.

To assess the relationship between total intake of vitamin A, β-carotene, and retinol in the diet and the risk of cardiovascular disease, three multivariate Cox proportional hazards regression models were established. Model 1 was unadjusted. Model 2 was adjusted for age, sex, regions, district, education, and Model 3 was adjusted for age, sex, regions, district, education, smoking, alcohol, income, BMI classification, physical activity, total energy, total protein, total carbohydrates, total dietary fiber, sodium to potassium intake ratio, calcium, zinc intake. Missing covariates were imputed using the MICE package in R Studio 4.2.3 software^30^, employing the Predictive Mean Matching (PMM) imputation method with five iterations to generate datasets for education (0.9%), individual income level (4.8%), body mass index (0.3%), and physical activity (0.3%). The linear trends were tested by assigning a median value to each quintile of the vitamin A intake. Moreover, the dose-response associations of dietary total vitamin A, β-carotene, retinol intake and the risk of CMM were examined using restricted cubic spline regression with five knots (Three knots were used in retinol intake and the risk of CMM), adjusted for the confounding variables mentioned above.

Stratified analyses were also performed according to age (<44/≥44), sex (male/female) and subcategories of CMM. To investigate whether the associations between dietary total vitamin A, β-carotene, retinol intake and the risk of CMM by these stratification variables, the potential effect modification was examined using the interaction models. In addition, we excluded participants who developed CMM during the first 2 years of follow-up to perform an assessment of the robustness of the results. All analyses were performed using R (version 4.3.2). All statistical tests were two-tailed and considered significant at *p* < 0.05.

## Results

### Characteristics of study participants

This study included a total of 13,603 participants, consisting of 6,534 males (48.0%) and 7,069 females (52.0%). The mean baseline age of the participants was 43.9 (SD 14.8). The median dietary intake of total vitamin A, β-carotene, and retinol is 530.5 (301.0, 998.2), 2602.0 (1418.8, 4730.4), and 110.4 (44.1, 288.7) μg RE/d. Table 1 shows the main baseline characteristics of participants in the vitamin A intake quintile group. Compared to the first group, participants in the highest intake group were more likely to reside in the northern region, have a higher proportion of urban residents, possess higher levels of education and income, and have a higher likelihood of alcohol consumption. In terms of dietary nutrient intake, the intake of all nutrients increased in the quintile groups, while the ratio of sodium to potassium intake showed a decreasing trend.

### Associations between vitamin A intake and cardiometabolic multimorbidity

**Figure 2:**
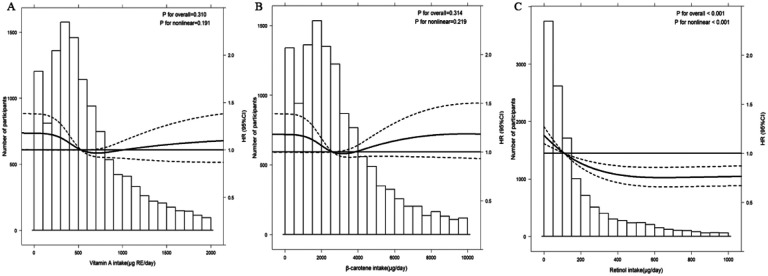
Cubic spline curves for the associations between intakes of total vitamin A (A), β-carotene (B) and retinol (C) with CMM risk. HR (95% CI) was based on a mixed-effects Cox proportional hazards model adjusted for age, sex, region, area, education, smoking, alcohol consumption, income, BMI classification, physical activity, total energy, total protein, total carbohydrates, total dietary fiber, sodium-to-potassium intake ratio, calcium, zinc intake. Histogram showing the distribution of intake of vitamin A, β-carotene, and retinol.

During a median follow-up period of 9.0 years, we ascertained 1050 incident cases of CMM. Table 2 shows the association between vitamin A intake and CMM risk. After adjusting for all covariates, the multivariate-adjusted HRs and 95% CIs from lowest to highest vitamin A intake were 1.00 (ref), 0.59 (0.48–0.73), 0.82 (0.67–1.00), 0.66 (0.54–0.81), and 0.80 (0.64–0.99), revealing a negative association, but the *p*-trend value (0.733) showed a non-significant linear trend. Therefore, we further analyzed the nonlinear relationship based on Model 3 by setting intake as a continuous variable and using restricted cubic spline. In Figure 2A the association between vitamin A intake and CMM risk appeared to be U-shaped, but not significantly (*p* non-linear 0.191).

### Association between β-carotene intake and cardiometabolic multimorbidity

A similar relationship was found in β-carotene Intake. When using the first quintile (Q1) as reference, the multivariable-adjusted HRs (95% CI) of Q2 to Q5 for CMM risk were 0.62 (0.50–0.77), 0.79 (0.65–0.96), 0.68 (0.56–0.84) and 0.82 (0.66–1.02), respectively, *p* for trend 0.871 (Table 2). We used a restricted cubic spline method to construct a visual model, confirming the U-shaped relationship between β-carotene intake and the risk of CMM, but not significantly (*p* non-linear 0.219, Figure 2B).

### Associations between retinol intake and cardiometabolic multimorbidity

In the analysis of retinol intake, the risk of CMM was consistently lower in Q2–Q5 than in Q1 in all models. fully adjusted HRs (95% CI) were 0.70 (0.57–0.85), 0.66 (0.54–0.80), 0.66 (0.54–0.81), 0.59 (0.48–0.73), respectively, *p* for trend 0.001 (Table 2). Spline models with fully adjusted covariates were constructed to profile a more direct relationship between retinol intake and cardiometabolic multimorbidity. As depicted in Figure 2C the results of multivariate Cox regression with restricted cubic spline (3 knots) found an L-shaped relationship (*p* non-linear<0.001). With an increase in retinol intake, the risk of CMM shows a decreasing trend until it stabilizes at around 500 μg/d.

### Subgroup analysis

To further explore the potential influencing factors of the relationship between vitamin A intake and CMM, we first conducted a Stratified analysis by age and gender. As can be seen in Table 3, age may have altered the relationship between vitamin A intake and CMM risk due to interaction effects, which can be more cautiously interpreted to mean that the negative association between vitamin A intake and CMM risk may be stronger in older subjects compared with younger subjects (<44 vs ≥44 years, *P*-interaction=0.049), and the same was seen in β-carotene and Retinol intake showed the same. Secondly, for male patients, the protective effects of vitamin A and β-carotene are retained, but Retinol was found to still play a role in reducing CMM risk in certain groups. In the terms of female patients, whether it is β-carotene, Retinol, or total vitamin A intake, the higher the intake compared to the low intake group, the lower the risk of CMM. However, the interaction was not significant.

**Supplemental Figure 1** shows the different groups of CMM. Among all participants, the top 5 groups were hypertension and diabetes (4.04% of the total population), hypertension and cardiovascular disease (1.24% of the total population), hypertension and stroke (1.13% of the total population), hypertension, diabetes, and cardiovascular disease (0.54% of the total population), and hypertension, diabetes, stroke (0.35% of the total population). There still exists an association between vitamin A and different combinations of CMM, although not significant in some categories (**Supplemental Table 1**). In order to minimize reverse causality as much as possible, after excluding patients who developed CMM during the first two years of follow-up, the analysis results were consistent with the main analysis results (**Supplemental Table 2**).

## Discussion

This study is grounded in a national population-based prospective cohort, utilizing information from 1997–2015, involving 13,603 subjects. According to our knowledge, this study is the first to report the association between different dietary sources of vitamin A and CMM, as well as different CMM clusters. We observed that both total dietary vitamin A, β-carotene and retinol intake, were significantly negatively associated with the risk of CMM.

In Stratified analyses, we observed that intake of more vitamin A may have a greater effect on reducing the risk of CMM in patients aged ≥ 44 years. The same is true for gender, where the beneficial effect of dietary vitamin A intake on CMM prevention may be more pronounced in female patients compared with male patients. Unfortunately, the protective effect of the CMM was preserved when analyzing different clusters of the CMM, especially the groups that included cardiovascular disease, which may be influenced by the small number of cardiovascular disease cases, and a larger population should be included in future studies for analysis. Furthermore, we observed a ceiling effect between retinol intake and the incidence of CMM, where the risk of developing CMM decreased as retinol intake increased, but eventually plateaued.

Research on the cardiometabolic effects of diets with high antioxidant capacity has been in the spotlight to date, and previous studies have reported extensively on the effects of vitamin A on cardiometabolic health at the level of serum concentrations, supplements, and dietary intake, respectively, but very few studies have examined the effects of dietary intake of vitamin A on the risk of CMM. According to previous reports, many studies have yielded results that increased intake of vitamin A is strongly associated with a reduced risk of hypertension^31–33^, diabetes^34^, cardiovascular disease^35,36^, and stroke^21^, and that vitamin A has beneficial cardiovascular effects by attenuating lipid peroxidation and free radical-induced damage^37^. Chi-Ho Lee and colleagues used data from the Hong Kong Cardiovascular Risk Factor Prevalence Study (CRISPS) to reported that intake of vitamins A through diet rather than supplementation not only did not increase cardiovascular events but also resulted in a lower risk of adverse cardiovascular outcomes after a median follow-up of 22 years (HR 0.68, 95%CI 0.53–0.88). A prospective cohort that systematically evaluated the relationship between 29 nutrients and CVD reported that dietary vitamin A intake was significantly negatively associated with CVD (HR 0.70, 95%CI 0.54–0.91). We added to the above evidence by directly examining the association between dietary vitamin A intake and CMM.

While vitamin A has received widespread attention, carotenoids, as the main components of vitamin A, not only possess the highest vitamin A activity^38^, but blood concentrations of carotenoids are also considered biomarkers of fruit and vegetable intake^39,40^, making them a focal point in research. Despite numerous studies exploring the association between carotenoids and cardiac metabolism, consensus has not yet been reached on the results. A meta-analysis of 69 prospective studies showed a non-linear relationship between total dietary carotenoid intake and cardiovascular disease (p non-linearity = 0.002), with most risk reductions ranging from 4,000 to 6,000 μg/d^41^. In a meta-analysis that included 10 trials and 16 reports, it was shown that slightly increased cardiovascular morbidity was observed when β-carotene supplementation was given alone (RR 1.04, 95% CI: 1.00–1.08)^42^. When people obtain natural β-carotene through the intake of plants and fruits, the bioavailability may be reduced due to changes in cell wall structure during food processing, as well as interactions with other dietary components and phytochemicals in the gastrointestinal tract^38,43^. Direct provision of supplemental intake through interventions may improve utilization, but it is also important to consider that the tolerable upper intake level may be influenced by the nutritional status and cardiovascular risk of the baseline population^42^. Excessive intake of β-carotene can lead to excessive depletion of free radicals, which can adversely affect cardiovascular health^44^. This may partly explain the inconsistent results between clinical trials and observational studies. In addition, Yi-Wen Jiang found an association between high dietary β-carotene intake and low risk of type 2 diabetes mellitus in 77,643 subjects from the West by integrating the results of six cohort studies (RR 0.78, 95%CI 0.70–0.87)^45^. A meta-analysis of stroke also reported that increased levels of β-carotene were more effective in reducing stroke risk at higher ages (log OR −0.61, 95%CI −1.09; −0.12)^21^. This corroborates with the observation of a negative association between dietary intake of β-carotene and CMM in our current study.

## Conclusions

In conclusion, our study suggests that dietary intake of vitamin A, β-carotene, and retinol is negatively associated with the risk of CMM, and that this negative association is stronger in age ≥ 44 years and in the female population. Among these, there was a ceiling effect on the protective effect of retinol intake on CMM.

## Figures and Tables

**Table 1:** Baseline characteristics of the study population according to Vitamin A intake quintiles and cumulative mean nutrient intake^1^

Characteristics	Quartile					*P*-value
	Q1 (N=2721)	Q2 (N=2720)	Q3 (N=2721)	Q4 (N=2720)	Q5 (N=2721)	
Age, years	45.5±15.9	42.4±15.2	42.5±14.5	44.0±14.3	44.9±13.7	0.001
Male, n (%)	1296 (47.6)	1273 (46.8)	1300 (47.8)	1337 (49.2)	1328 (48.8)	0.417
North, n (%)	1440 (52.9)	1806 (66.4)	1797 (66.0)	2042 (75.1)	1787 (65.7)	0.001
Urban residence, n (%)	979 (36.0)	776 (28.5)	1002 (36.8)	1322 (48.6)	1279 (47.0)	0.001
Education, n (%)						0.001
Illiteracy	731 (26.9)	672 (24.7)	602 (22.1)	544 (20.0)	484 (17.8)	
Primary school	522 (19.2)	558 (20.5)	556 (20.4)	490 (18.0)	492 (18.1)	
Middle school	807 (29.7)	887 (32.6)	829 (30.5)	838 (30.8)	822 (30.2)	
High school or above	661 (24.3)	603 (22.2)	734 (27.0)	848 (31.2)	923 (33.9)	
Smoking status, n (%)						0.027
Current smoker	818 (30.1)	839 (30.8)	809 (29.7)	809 (29.7)	812 (29.8)	
Former	61 (2.2)	27 (1.0)	35 (1.3)	37 (1.4)	43 (1.6)	
Never	1842 (67.7)	1854 (68.2)	1877 (69.0)	1874 (68.9)	1866 (68.6)	
Alcohol drinking, n (%)	1181 (43.4)	1227 (45.1)	1290 (47.4)	1279 (47.0)	1280 (47.0)	0.013
Individual Income level, n (%)						0.001
Low	723 (26.6)	917 (33.7)	738 (27.1)	545 (20.0)	478 (17.6)	
Medium	593 (21.8)	720 (26.5)	751 (27.6)	696 (25.6)	641 (23.6)	
High	603 (22.2)	602 (22.1)	719 (26.4)	772 (28.4)	774 (28.4)	
Very high	802 (29.5)	481 (17.7)	513 (18.9)	707 (26.0)	828 (30.4)	
Body mass index, kg/m^2^	23.8 (2.8)	23.7 (3.0)	23.7 (2.9)	23.7 (2.9)	23.7 (2.7)	0.597
Prevalent morbidity, n (%)					
Diabetes	164 (6.0)	196 (7.2)	213 (7.8)	240 (8.8)	231 (8.5)	0.001
hypertension	1512 (55.6)	1531 (56.3)	1432 (52.6)	1474 (54.2)	1445 (53.1)	0.030
Stroke	77 (2.8)	57 (2.1)	75 (2.8)	55 (2.0)	59 (2.2)	0.135
Cardiovascular disease	96 (3.5)	77 (2.8)	107 (3.9)	86 (3.2)	99 (3.6)	0.195
Dietary intake					
Vitamin A, μg RE/d	123.2 (0, 200.9)	342.3 (301.0, 386.5)	531.5 (478.2, 592.8)	846.0 (740.0, 998.1)	1932.1 (1459.7, 3069.5)	0.001
β-carotene, μg/d	530.2 (0, 967.5)	1731.5 (1469.9, 2010.6)	2763.6 (2426.1, 3141.4)	4418.2 (3751.3, 5242.3)	9128.1 (6954.2, 13664.0)	0.001
Retinol, μg/d	21.4 (0, 76.6)	89.1 (45.8, 187.7)	115.7 (58.9, 253.2)	157.9 (79.6, 390.4)	262.9 (111.6, 905.6)	00.001
Energy, Kcal/d	1896.3 (1502.7, 2277.7)	2064.6 (1769.8, 2392.4)	2098.7 (1822.8, 2401.2)	2124.8 (1802.7, 2444.5)	2154.4 (1839.2, 2496.5)	0.001
Protein, g/d	58.4 (47.1, 71.2)	62.3 (52.8, 73.0)	64.5 (55.5, 74.1)	67.3 (57.0, 78.9)	70.3 (59.7, 81.4)	0.001
Carbohydrate, g/d	260.2 (187.8, 339.9)	303.3 (245.1, 362.1)	296.1 (246.2, 354.4)	287.1 (231.1, 342.3)	281.8 (227.9, 343.7)	0.001
Dietary fiber, g/d	10.5 (1.8, 24.9)	18.9 (8.8, 31.9)	22.8 (10.5, 35.5)	29.2 (16.1, 40.9)	42.7 (28.6, 63.8)	0.001
Sodium to potassium ratio	3.1 (1.7, 11.1)	2.4 (1.5, 3.4)	2.0 (1.3, 3.1)	1.7 (1.2, 2.5)	1.2 (0.8, 1.7)	0.001
Calcium, mg/d	319.9 (50.5, 723.1)	540.8 (307.4, 903.0)	721.7 (357.6, 1064.1)	1020.1 (609.9, 1401.7)	1590.0 (1118.6, 2275.3)	0.001
Zinc, mg/d	9.9 (1.1, 18.6)	14.8 (9.6, 23.4)	19.2 (11.1, 27.3)	26.0 (16.1, 34.5)	39.8 (27.6, 64.6)	0.001

1 Variables are presented as mean ± SDs, median (IQR) or N (%). Comparison according to quintiles of vitamin A intake was performed by χ^2^ test for categorical variables, ANOVA or Kruskal-Wallis test for continuous variables.

**Table 2:** The relationship between cumulative mean intake of vitamin A, β-carotene and retinol and cardiometabolic multimorbidity risk^1^

Vitamin A intake	Quartile					*P*-trend^2^
	Q1 (N=2721)	Q2 (N=2720)	Q3 (N=2721)	Q4 (N=2720)	Q5 (N=2721)	
Vitamin A						
Median intake (μg RE/d)	123	342	531	846	1932	
Cases/person-years	210 / 257,952	166 / 353,292	230 / 354,744	218 / 342,156	226 / 326,772	
Model 1^3^	1.00 (ref)	0.54 (0.44–0.66)	0.74 (0.62–0.90)	0.74 (0.61–0.89)	0.81 (0.67–0.98)	0.502
Model 2^4^	1.00 (ref)	0.58 (0.47–0.71)	0.80 (0.67–0.97)	0.64 (0.53–0.77)	0.75 (0.62–0.91)	0.386
Model 3^5^	1.00 (ref)	0.59 (0.48–0.73)	0.82 (0.67–1.00)	0.66 (0.54–0.81)	0.80 (0.64–0.99)	0.733
β-carotene						
Median intake (μg/d)	530	1732	2764	4418	9128	
Cases/person-years	207 / 250,584	168 / 350,868	226 / 361,524	218 / 348,564	231 / 323,376	
Model 1^3^	1.00 (ref)	0.54 (0.44–0.66)	0.70 (0.58–0.84)	0.71 (0.58–0.86)	0.82 (0.68–0.99)	0.334
Model 2^4^	1.00 (ref)	0.61 (0.49–0.74)	0.78 (0.64–0.94)	0.65 (0.54–0.79)	0.75 (0.62–0.90)	0.275
Model 3^5^	1.00 (ref)	0.62 (0.50–0.77)	0.79 (0.65–0.96)	0.68 (0.56–0.84)	0.82 (0.66–1.02)	0.871
Retinol						
Median intake (μg/d)	21	89	116	158	263	
Cases/person-years	221 / 257,964	183 / 339,468	219 / 350,856	242 / 349,536	185 / 337,092	
Model 1^3^	1.00 (ref)	0.59 (0.49–0.72)	0.68 (0.57–0.83)	0.76 (0.64–0.92)	0.61 (0.50–0.74)	0.007
Model 2^4^	1.00 (ref)	0.69 (0.57–0.84)	0.68 (0.57–0.83)	0.71 (0.59–0.85)	0.64 (0.53–0.78)	0.008
Model 3^5^	1.00 (ref)	0.70 (0.57–0.85)	0.66 (0.54–0.80)	0.66 (0.54–0.81)	0.59 (0.48–0.73)	0.001

1 Values are HRs (95% CIs) based on mixed-effects Cox proportional hazards models.

2 *P*-trend was detected by assigning a median value to each quintile of vitamin A intake.

3 Model 1: Unadjusted.

4 Model 2: Adjusted for age, sex, regions, district, education.

5 Model 3: Model 2 + smoking, alcohol, income, BMI classification, physical activity, total energy, total protein, total carbohydrates, total dietary fiber, sodium to potassium intake ratio, calcium, zinc intake.

**Table 3:** Subgroup analysis for the association between vitamin A, β-carotene and retinol and CMM risk^1^

Subgroup	Age					Sex				
	≥44	*P*-value	≥44	*P*-value	*P*-interaction	Male	*P*-value	Female	*P*-value	*P*-interaction
Vitamin A					0.049					0.145
Q1	1		1			1		1		
Q2	0.98 (0.56–1.72)	0.954	0.55 (0.44–0.69)	<0.001		0.79 (0.58–1.06)	0.117	0.46 (0.34–0.62)	<0.001	
Q3	1.37 (0.81–2.31)	0.239	0.69 (0.56–0.85)	0.001		0.90 (0.67–1.22)	0.504	0.69 (0.53–0.90)	0.007	
Q4	0.96 (0.55–1.68)	0.876	0.63 (0.50–0.79)	<0.001		0.77 (0.57–1.06)	0.105	0.55 (0.42–0.73)	<0.001	
Q5	1.09 (0.59–2.01)	0.785	0.72 (0.57–0.92)	0.009		1.04 (0.75–1.44)	0.822	0.62 (0.45–0.84)	0.002	
β-carotene					0.099					0.184
Q1	1		1			1		1		
Q2	1.17 (0.67–2.07)	0.581	0.58 (0.46–0.73)	<0.001		0.79 (0.58–1.07)	0.13	0.48 (0.36–0.65)	<0.001	
Q3	1.25 (0.72–2.18)	0.432	0.69 (0.56–0.86)	0.001		0.86 (0.64–1.17)	0.34	0.72 (0.55–0.93)	0.013	
Q4	1.19 (0.67–2.10)	0.552	0.66 (0.52–0.82)	<0.001		0.80 (0.58–1.09)	0.151	0.56 (0.43–0.74)	<0.001	
Q5	0.99 (0.51–1.92)	0.987	0.77 (0.60–0.99)	0.042		1.10 (0.79–1.53)	0.579	0.62 (0.46–0.85)	0.02	
Retinol					0.266					0.487
Q1	1		1			1		1		
Q2	0.89 (0.52–1.52)	0.662	0.55 (0.44–0.69)	<0.001		0.83 (0.62–1.11)	0.202	0.55 (0.42–0.73)	<0.001	
Q3	0.75 (0.43–1.30)	0.311	0.63 (0.51–0.78)	<0.001		0.73 (0.55–0.98)	0.038	0.53 (0.41–0.70)	<0.001	
Q4	0.92 (0.53–1.59)	0.759	0.55 (0.44–0.68)	<0.001		0.77 (0.57–1.03)	0.078	0.51 (0.39–0.67)	<0.001	
Q5	0.77 (0.43–1.36)	0.368	0.53 (0.42–0.66)	<0.001		0.62 (0.45–0.85)	0.003	0.52 (0.39–0.69)	<0.001	

1 HR (95% CI) was based on a mixed-effects Cox proportional hazards model adjusted for age, sex, region, area, education, smoking, alcohol consumption, income, BMI classification, physical activity, total energy, total protein, total carbohydrates, total dietary fiber, sodium-to-potassium intake ratio, calcium, zinc intake.

## Data Availability

Data can be accessed via https://www.cpc.unc.edu/projects/china/data.
